# A noise-suppressing neural network approach for upper limb human-machine interactive control based on sEMG signals

**DOI:** 10.3389/fnbot.2022.1047325

**Published:** 2022-11-03

**Authors:** Bangcheng Zhang, Xuteng Lan, Gang Wang, Zaixiang Pang, Xiyu Zhang, Zhongbo Sun

**Affiliations:** ^1^Department of Mechatronical Engineering, Changchun University of Technology, Changchun, China; ^2^Department of Industrial Engineering, Changchun University of Technology, Changchun, China; ^3^Department of Control Engineering, Changchun University of Technology, Changchun, China

**Keywords:** SEMG signal, zeroing neural network, motion intention recognition, human-machine interaction controller, noise-suppressing

## Abstract

The use of upper limb rehabilitation robots to assist the affected limbs for active rehabilitation training is an inevitable trend in the field of rehabilitation medicine. In particular, the active motion intention-based control of the upper limb rehabilitation robots to assist subjects in rehabilitation training is a hot research topic in human-computer interaction control. Therefore, improving the accuracy of active motion intention recognition is the premise of the human-machine interaction controller design. Furthermore, there are external disturbances (bounded/unbounded disturbances) during rehabilitation training, which seriously threaten the safety of subjects. Thereby, eliminating external disturbances (especially unbounded disturbances) is the difficulty and key to the human-machine interaction control of the upper limb rehabilitation robots. In response to these problems, based on the surface electromyogram signal of the human upper limb, this paper proposes a fuzzy neural network active motion intention recognition method to explore the internal connection between the surface electromyogram signal of the human upper limb and active motion intention, and improve the real-time and accuracy of recognition. Based on this, two types of human-machine interaction controllers, which can be called as zeroing neural network controller and noise-suppressing zeroing neural network controller are designed to establish a safe and comfortable training environment to avoid secondary damage to the affected limb. Numerical experiments verify the feasibility and effectiveness of the proposed theories and methods.

## 1. Introduction

The global situation of population aging tends to be increasingly serious. Several countries such as the United States, China, the United Kingdom, and Japan have become the countries with the largest number of elderly people in the world. At the same time, the number of physical disabilities caused by stroke, spinal cord injury, and brain trauma is increasing rapidly. Among them, stroke is the main disease that causes local skeletal muscle motor dysfunction in people's upper limbs. Stroke has the characteristics of high disability rate, obvious younger trend, and great harm, which seriously endangers the physical and mental health of patients, and brings a heavy economic burden to patients' families and society (Huo et al., 2016; Young and Ferris, 2017; Venkatesh et al., 2019). Because the injured limb has a certain active movement ability, it can actively drive the bones and muscles of the injured limb for rehabilitation training activities (Fournier et al., 2018; Orekhov et al., 2020). However, the injured limb after stroke is often accompanied by abnormal muscle activities such as motor incoordination, spasm, fatigue, and tremor, which are prone to humans. Therefore, the phenomenon of machine confrontation will cause secondary injury to the affected limb and bring huge safety hazards to patients' rehabilitation training.

The upper limb rehabilitation robot assists in rehabilitation training activities of the affected upper limb after stroke. Repetitive task-oriented training is the goal to formulate rehabilitation treatment plans, which can improve the motor function of the affected limb, enhance sensory input, strengthen the brain's functional reorganization ability, and stimulate brain cells mobility to improve the motor function of the affected limb and the self-care ability of daily life. There are many algorithms have been developed to control the rehabilitation robots to assist subject movement in current research. For instance, a control method that combines proportional-derivative (PD) control, sliding mode control (SMC) and fuzzy logic control is developed to achieve high position tracking performance for the upper limb exoskeleton in Teng et al. (2020). However, in the process of rehabilitation training, the affected limb is often accompanied by uncoordinated motor function and poor control, which leads to human-machine confrontation and even secondary injury (Wu et al., 2018a,b). To ensure the smooth progress of rehabilitation training and avoid human-machine confrontation and its adverse consequences, the interactive control between the upper-limb rehabilitation robot and the affected limb must be indispensable in the process of rehabilitation training. Good human-machine interaction control will create a safe, natural and compliant rehabilitation training environment for patients, which can not only avoid secondary injury to the affected limb rehabilitation training due to the time-varying information of working conditions, but also promote the active participation of patients in rehabilitation. The confidence of training greatly improves the rehabilitation effect (Zhang and Cheah, 2015; Aach et al., 2016; Deng et al., 2020). Due to the strong randomness of human movement behavior, the control system of upper limb rehabilitation robot should have the ability to quickly adjust the system parameters, which can provide the patient with the best rehabilitation exercise mode in real time and help the affected limb to restore normal movement functions.

Real-time and accurate recognition of the current state and movement intention of the affected limb can help patients achieve effective information interaction between the affected limb and the upper limb rehabilitation robot. Therefore, accurate recognition of the movement intention of the affected limb is the prerequisite for human-machine interaction control, and the design of an effective human-machine interaction control method is the key-point to realize the coordinated movement of the affected limb and the upper limb rehabilitation robot. Park et al. developed an adaptive impedance control method for upper limb exoskeleton robots using biomechanical signals (Park et al., 2018). Chen et al. proposed an auxiliary control system embedded with force/torque sensors, and demonstrated the inherent mapping relationship between robot and human, and analyzed the mapping relationship of rehabilitation patients in different modes (Chen et al., 2016). The results of rehabilitation training show that this method has made a certain contribution in clinical application. Moreover, Zhao et al. proposed a critical damping controller for the cascade-series elastic driver control structure, which gave a new impedance performance index “Z zone” used to quantify the achievable impedance amplitude (Zhao et al., 2018). The feasibility and effectiveness of the proposed method are verified by simulation and experiment. However, the collection of the biomechanical signals lags behind the human movement, which is not flexible and convenient.

Compared with biomechanical signals, the physiological signals (surface electromyographic (sEMG) signals), with higher sensitivity and resolution are more suitable for intention recognition. Cene proposed a human-machine interaction control method based on the sEMG signals and adaptive frequency oscillator in Cene and Balbinot (2020). Through numerical simulation and plane parallel structure upper limb rehabilitation robot platform experiment, the synchronous active rehabilitation training method was verified feasibility and effectiveness. Furthermore, the human-robot cooperative control method based on sEMG signals is developed to drive the pneumatic upper limb exoskeleton to act according to the wearer's motion intentions (Liu et al., 2020). For pathological tremor, motion sensors are usually used to detect, activate the stimulus signal transmitted by the muscle within the range of the motion threshold to resist vibration and vibration, and achieve active rehabilitation training with different auxiliary exercise levels. However, it is inevitable that data acquisition is affected by different measurement noises during the use of sensors, because noise is inevitable and always exists in the actual system. Beyond that, the uncertainty of the model also brings great challenges to the research on human-computer interaction control of the upper limb. Peng et al. proposed a sensorless control strategy that integrates radial basis function neural network, force estimation, and admittance control in Peng et al. (2020), which ensures that Baxter robots can interact with unknown environments under input constraints. The adaptive neural controller can ensure that the tracking performance and tracking error of the system are in the neighborhood of the stable point. By estimating the external torque exerted by the end effector, the admittance control method is adopted to adapt the trajectory to achieve self-adaptability. Kim et al. proposed a novel time delay control strategy, which significantly improves the performance of interactive force control and effectively solves the problems of uncertainty due to physical human-machine interaction systems and inaccurate force control (Kim and Bae, 2016). Furthermore, Brahim proposed an inversion method combined with time delay estimation, which solves the joint connection and its constraint problems of dynamic system uncertainty caused the time delay errors (Brahim et al., 2018). The designed feed-forward loop directly couples the output of the time delay estimator to the adaptive tracking control input, which can ensure accurate tracking of the control trajectory, and is robust to uncertainties and unpredictable external forces, and can adapt to the changes in parameters. However, the external disturbances are frequently regarded as a bounded disturbances in current studies. Actually, the external disturbances are unknown noises, and the noises strengths are unpredictable. That is to say, the external disturbances may be unbounded noises. In summary, as the actual demand for the performance of rehabilitation robots continues to increase, human-machine interaction control strategies emerge in endlessly, but the existing methods still have some problems, for example, 1) The accuracy of intention recognition needs to be further improved to ensure the safety of subjects in the rehabilitation process and enhance sensory input. 2) The external disturbances (bounded/unbounded disturbances) have great influence on the model. It is significantly important to construct a noise-tolerant control method based on a human-machine model, which can create a safe, comfortable and supple rehabilitation training environment for the injured limb.

Thereby, to resolve the above problems, the fuzzy neural network is proposed to identify the active motion intention of the subjects based on the multi-channel sEMG signals and takes it as the expected trajectory of the subjects. In addition, considering the interference of external disturbances (bounded/unbounded disturbances) on the model, a human-machine interaction controller that can suppress different external disturbances is analyzed and developed based on the active intention of the subjects in this paper. In summary, the main contributions of this paper are concluded as follows:

(1) Based on the multi-channel sEMG signals, the active motion intention of the subjects is effectively identified, and the effective information interaction between the subjects and the upper limb rehabilitation robot is realized, which lays the foundation for the research of human-machine interaction controller.
(2) Based on the active motion intention of the subjects, an noise-suppressing zeroing neural network (NSZNN) controller is developed to eliminate different external disturbances, which can avoid unnecessary damage caused by noise/disturbance during rehabilitation training.
(3) Experimental results demonstrate that the proposed human-machine interactive controller has the advantage over the classical proportional-integral-derivative (PID) controller, gradient neural network (GNN) controller and zeroing neural network (ZNN) controller in terms of prediction accuracy and noise tolerant properties under different noise-polluted conditions.

The chapters of this paper are arranged as follows: Section 2 is the recognition of upper limb active movement intention, and a fuzzy neural network is constructed to realize the upper limb active movement intention recognition; Section 3 is the design of the human-machine interaction controller, which constructs two types of human-machine interaction controllers to ensure the absolute safety and smooth interaction of the affected limb, and improves the interaction efficiency and rehabilitation effect; Section 4 is the experimental results verify the feasibility and effectiveness of the proposed algorithm; Section 5 discusses the summarize of this paper.

## 2. Active movement intention recognition for upper limbs

### 2.1. Data collection and preprocessing

**Figure 1:**
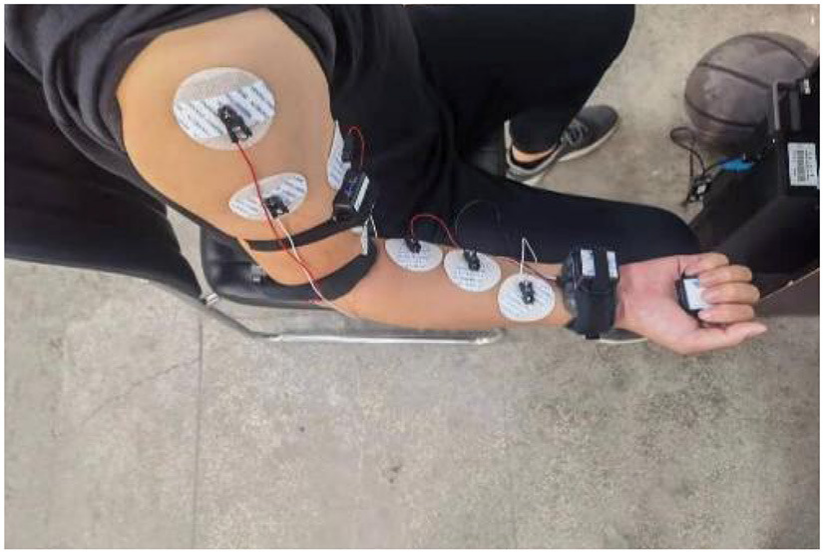
The environment of multi-channel sEMG and angle signals for upper limb.

The sEMG signal can reflect the activity state of the muscle during exercise to a certain extent (Wang et al., 2019; Chai et al., 2021; Wei et al., 2021). Through the corresponding time and frequency domain analysis, the time and frequency domain characteristics and the corresponding muscle characteristics and movement correlation can be obtained, and the muscle function state of the human body during exercise can be obtained. Through experiments, the corresponding sEMG signals of the deltoid muscle, biceps brachii, and pronator teres muscle of the upper limbs of the human body were obtained through experiments, and the angles of the three joints of the shoulder, elbow and wrist were measured and recorded at the same time. In order to ensure the accuracy of the data obtained during the measurement, the MP160 electromyographic signal acquisition device developed by the American BIOPAC company is used to obtain the electromyographic information corresponding to the muscles of the human upper limbs during exercise, and the WT901C485 angle sensor produced by China Huitong Company is used to collect the angles of the upper limbs three joints. During data collection, the subject is required to perform the corresponding upper limb flexion and extension exercises, and the measured data at this time is used as the pre-input data of the model, as shown in Figure 1.

**Figure 2:**
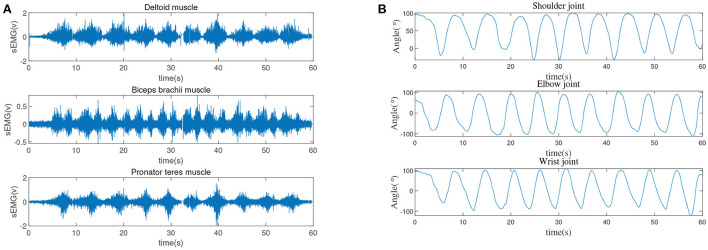
The acquisition of the multi-channel sEMG signals and multi-joint angles, **(A)** the original sEMG signals of the upper limbs, **(B)** the movement angles of the three joints of the upper limbs.

The signal collection process is susceptible to interference from the external environment. In order to avoid external interference as much as possible, it is necessary to perform corresponding dehairing treatment on the skin surface of the person being measured, and apply appropriate electrode gel to reduce the impedance between the electrode and the skin. The silver chloride electrode pads used in the sEMG collection process will also interfere with the collected data. Therefore, the distance between two adjacent electrode pads is selected as 2 cm in this experiment. The specific measurement results are displayed in Figure 2.

sEMG signal is a weak electrical signal generated during muscle contraction. It has high randomness and instability, which is easily affected by the external environment. For example, the tester's skin, hair and sweat are also easily affected by power frequency interference caused by the power system (Han et al., 2015; Nougarou et al., 2019; Huang et al., 2021). In addition, the position of the electrode patch too close will also cause superimposed interference on the signal collected by the adjacent electrode patch. According to the muscle and skeletal structure of the human upper limbs, the flexion and extension movement of the shoulder joint mainly relies on the anterior deltoid and posterior deltoid. In detail, the elbow joint corresponds to the biceps brachii and triceps brachii, and the wrist joint depends on the extensor carpi radialis and flexor carpi radialis. Therefore, the sEMG signals of six muscles, which includes the anterior deltoid, posterior deltoid, biceps brachii, triceps brachii, extensor carpi radialis and flexor carpi radialis need to be collected. Based on this, how to filter the noise becomes the primary task after the signal acquisition. The energy of the sEMG signal is higher than the level of electronic noise, and the frequency range is between 0 and 500 Hz, mainly distributed between 50 and 150 Hz. The lowest frequency of the sEMG signal is 20 Hz. This article uses a band-pass filter with a low cut-off frequency of 20 Hz and a high cut-off frequency of 500 Hz to filter out some of the noise factors during signal acquisition. In addition, the commonly used power frequency interference is set as 50 Hz, which is within the range of the available signal energy concentration, therefore, a 50 Hz notch filter is used to eliminate power frequency interference. Besides, Figures 2A,B show that the raw sEMG signals and joint angle collected in 60 s, specifically, the sampling frequency of Biopac system and IMU module are 2 kHz and 100 Hz respectively, that is to say, the sampling time of Biopac system and IMU module are 0.0005 s and 0.01 s, respectively. To obtain sEMG signals and joint angle, in this experiment, a healthy male (27 years old, 182 cm, 80 kg) volunteered for upper limb flexion and extension exercises in the sagittal plane.

**Figure 3:**
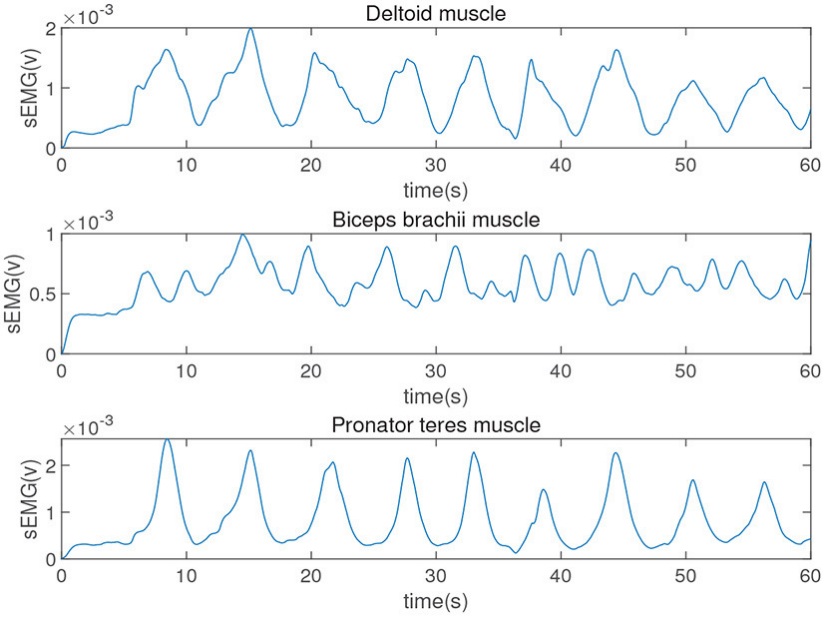
The sEMG signals of the upper limb after processing.

Through the above-mentioned filtering steps, the sEMG signal after the corresponding noise filtering can be obtained. However, the amplitudes of sEMG signals are random in nature, and the signal vibrates very frequently at the zero point Zhang et al. (2012). Therefore, to more clearly present the changing process of the amplitude, the following full-wave rectification technology is exploited to process sEMG signals.


(1) $$\begin{array}{l} {\text{sEMG}}_{\text{rec}}\left(n\right)=|\text{sEMG}\left(n\right)| \end{array}$$

where sEMG(*n*) is the original sEMG signal, and is the sEMG signal after full-wave rectification. By comparing the sampling rate of the actual joint angle signal, it can be seen that the sampling rate of the sEMG signal collected in the experiment is too large, so this article performs sub-sampling on the obtained sEMG signal,


(2) $$\begin{array}{l} {\text{sEMG}}_{\text{ss}}\left(n\right)=\frac{1}{N}\Sigma_{i=nN-N+1}^{nN}|\text{sEMG}\left(i\right)| \end{array}$$

where *N* is the number of sampling times of the sEMG signal, and is the *N*th sEMG signal after sub-sampling. After digital filtering, full-wave rectification and sub-sampling, the envelop of the sEMG signals still vibrate very much. Generally, a low-pass filter such as Butterworth or Bessel filters can be employed to smooth the envelope of the sEMG signals Zhang et al. (2012). Furthermore, in view of the outward manifestation of the muscle contraction such as joint angle changing presents low-frequency characteristics, the following first- order low-pass Butterworth filter with cut-off frequency 5 Hz is exploited to filter the high frequencies.


(3) $$\begin{array}{l} |H\left(w\right){|}^{2}=\frac{1}{1+{\left(\frac{w}{w_{\text{c}}}\right)}^{2n}} \end{array}$$

where *n* = 1 is the order of the filter and *w*_c_ = 5 Hz is the cutoff frequency. Integrating all the above-mentioned signal processing processes, the processed sEMG signal can be finally obtained as shown in Figure 3.

### 2.2. Construction of the fuzzy neural network

The fuzzy neural network structure combines fuzzy inference mechanism and neural network prediction techniques. The structure of a fuzzy neural network includes an input layer, a membership function layer, a fuzzy rule layer and an output layer. Each layer has several neuron nodes, and adjacent nodes are connected by a certain weight ratio. The input and output nodes of the neural network are used to realize the input and output signals of the system. The hidden nodes represent the membership function and fuzzy rules.

The input of the adaptive neural network structure is the sEMG signal obtained from the signal processing link. The input data of the specific model is as follows:


(4) $$\begin{array}{l} x=\left[x_{1},x_{2},\cdot \cdot \cdot ,x_{t}\right],\;t=10000 \end{array}$$

Among them *x* is the processed muscle sEMG signals measured within 100s. In this paper, the three joints measured by the angle sensor are used as the expected output of the model to compare the prediction results of the adaptive fuzzy neural network (AFNN) model to further verify the reliability of the prediction model. The actual three joints measured are as follows:


(5) $$\begin{array}{l} \theta_{\text{act}}=\left[\theta_{1},\theta_{2},\cdot \cdot \cdot ,\theta_{t}\right],\;t=10000 \end{array}$$

where θ_act_ is the actual three joints measured in 100 s, and the sampling frequency is 100 Hz.

The established fuzzy neural network model is as follows:


(6) $$\begin{array}{l} y=\Sigma_{j=1}^{L}f_{j}\phi_{j}\left(\frac{\left||x-\mu_{j}|\right|}{\sigma_{j}}\right) \end{array}$$

where ϕ_*j*_ is the membership function set of the input variables *x*, μ_*j*_ is the center of the membership function, σ_*j*_ is the variance vector of the membership function, and *f*_*j*_ is the weight of the membership function ϕ_*j*_.

This paper selects the Gaussian function when the prediction result is optimal as the center of the membership function. The specific mathematical expression of the membership function is as follows:


(7) $$\begin{array}{l} \phi_{j}=e^{-{\left(\frac{x_{i}-\mu_{ij}}{\sigma_{i}j}\right)}^{2}} \end{array}$$

and


(8) $$\begin{array}{l} \mu_{ij}=\frac{1}{1+e^{\left(\frac{b_{ij}x_{ij}+c_{ij}^{2}}{\sigma_{i}j}\right)}} \end{array}$$

Combining the above formulas, the AFNN model can be expressed in the following form:


(9) $$\begin{array}{l} y=\frac{\Sigma_{j}^{L}f_{j}\Pi_{i}^{n_{j}}e^{-\left(\frac{x_{i}-\mu_{ij}}{\sigma_{ij}}\right)}}{\Sigma_{j}^{L}\Pi_{i}^{n_{j}}e^{-\left(\frac{x_{i}-\mu_{ij}}{\sigma_{ij}}\right)}} \end{array}$$

where 1 ≤ *n*_*j*_ ≤ *N* is the dimension of the membership function ϕ_*j*_, *N* is the dimension of the input variable, and *L* is the number of nodes in the fuzzy rule layer of the neural network. AFNN model usually combines the membership function in the model into fuzzy rules. The Takagi-Sugeno (T-S) fuzzy model is a nonlinear model with strong adaptive ability composed of fuzzy inference and fuzzy rules. The fuzzy regular expression is as follow:


(10) $$\begin{array}{l} R_{L}:\text{if}x_{1}=A_{1l},x_{2}=A_{2l},\cdot \cdot \cdot ,x_{k}=A_{kl},\text{then}{\;y}_{l}=P_{0l}\\ +P_{1l}x_{1}+\cdot \cdot \cdot ,+P_{Ml}x_{M} \end{array}$$

where *A*_*Ml*_ is the fuzzy set of the fuzzy neural network system; *P*_*Ml*_ is the parameter of the fuzzy system; *y*_*l*_ represents the output result obtained by the fuzzy rule.

### 2.3. Active movement intention recognition

**Figure 4:**
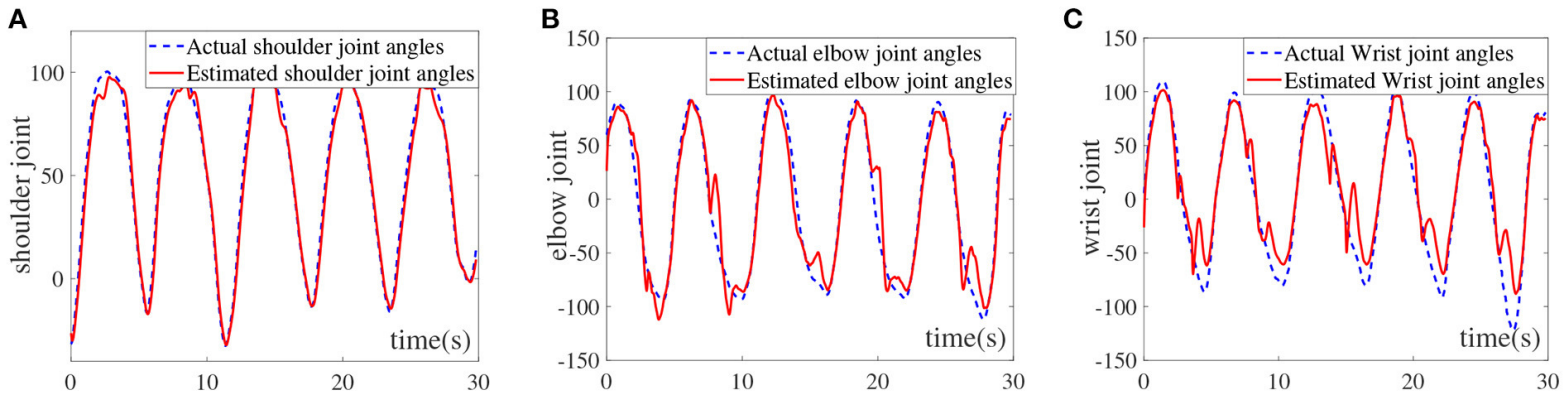
Comparison of predicted and actual values of the upper limb multi-joints, **(A)** shoulder joint, **(B)** elbow joint, and **(C)** wrist joint.

Using MATLAB2016a simulation software, the simulation experiment of AFNN prediction model algorithm is carried out based on the sEMG signals of the human upper limbs and the angle measurement data of joints, and obtain the comparison between the predicted value and the actual value of the joints, which is shown in Figure 4.

To verify the feasibility and validity of the proposed model, the root mean square error (RMSE) and coefficient of determination (*R*^2^) are exploited to quantify the accuracy of the model. Where, the RMSE can be computed as


(11) $$\begin{array}{l} RMS=\sqrt{\frac{1}{\Omega }\overset{\Omega }{\underset{1}{\sum }}\left(\theta_{i}^{a}-\theta_{i}^{e}\right)} \end{array}$$

where Ω is the total number of samples, $\theta_{i}^{a}$ is the collected joint angle by IMU system at *i*th, and $\theta_{i}^{e}$ is the estimated angle by CNN model at *i*th. The RMSE directly compares the estimated angle with the actual angle, which can intuitively reflect the estimation accuracy of the model. Furthermore, *R*^2^ can be calculated as follows:


(12) $$\begin{array}{l} R^{2}=1-\frac{\text{Var}\left(\theta^{a}-\theta^{e}\right)}{\text{Var}\left(\theta^{a}\right)} \end{array}$$

where θ^*a*^ the collected actual joint angle by IMU system, θ^*e*^ is the CNN model estimation, and Var(·) is the variance of the sample. Different from the RMSE, *R*^2^ is more robust to the numerical range of labels. The closer *R*^2^ is to 1, the better the model accuracy, and the *R*^2^ < 0 means that the model error is higher than the variance of the target value.

It can be seen from Figure 4A that the AFNN can be effectively used to estimate the shoulder joint angle. Although there are certain degree of deviations at 15, 20, and 27 s, the overall predicted trend of the shoulder joint conforms to the actual shoulder joint movement angle. The RMSE and *R*^2^ of the shoulder joint are calculated to be 4.8393 and 0.9883, respectively, which infers that AFNN can accurately estimate the angle of the shoulder joint. Furthermore, Figure 4B shows the elbow joint estimation results. It can be seen that the AFNN can also accurately estimate the elbow joint angle. The RMSE and *R*^2^ of the elbow joint are 8.6652 and 0.9751, respectively. It is worth noting that the valley value cannot reach the actual situation and the peak fluctuation situation occurs. This is because the elbow reached a critical range, the muscles may occur tremors resulting in increased estimation error. As displayed in Figure 4C, the estimation error of the wrist joint is relatively large, especially at the wave trough. Moreover, the RMSE and *R*^2^ of the wrist joint are 10.8265 and 0.9554, respectively, which proves that the AFNN can still be used to estimate the angle of the wrist joint. Based on the above-mentioned intention recognition results for the three joints of the upper limbs, it can be seen that the proposed AFNN algorithm is relatively accurate for the intention recognition of the upper limbs. The effective control of the upper limb rehabilitation robot is realized by designing a human-machine interaction controller to provide patients with a safe rehabilitation training environment. However, in order to realize human-machine interaction, high-precision intention recognition methods are indispensable. Therefore, the fuzzy neural network motion intention recognition algorithm constructed in this paper lays an algorithm framework for the design of interactive controllers.

## 3. Human-machine interaction controller based on ZNN and NSZNN

**Figure 5:**
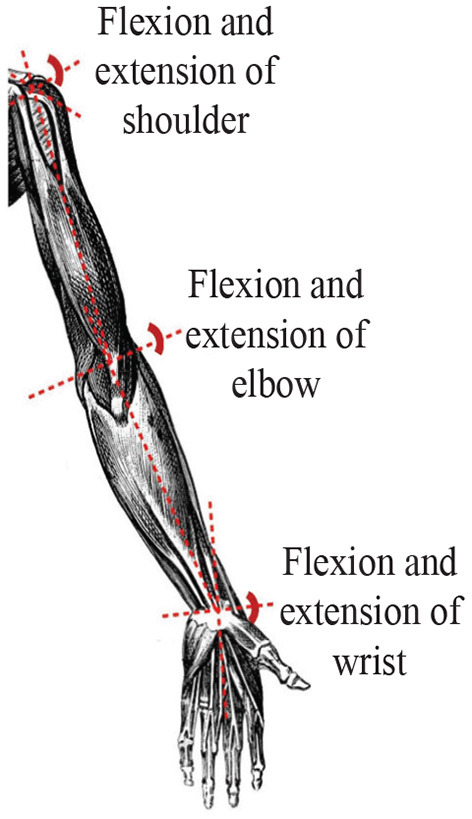
Human upper limb skeleton.

In this section, based on Lagrangian dynamics and the intention of active movement of the upper limb shoulder, elbow and wrist joints, a human-machine interaction controller is designed to effectively control the upper limb rehabilitation robot. Under the premise of human-machine information interaction, rehabilitation robots can drive patients to perform rehabilitation training. Recently, due to the conspicuous performances like adaptivity, distributed storage function, and parallel computing schemes, zeroing neural network-based (ZNN-based) models with superior robustness and effectiveness, which can be seen as a special case of recurrent neural network (RNN), have been widely utilized to solve zero finding problems (Jin et al., 2016, 2017, 2018, 2019; Sun et al., 2020, 2022). Based on the fuzzy neural network motion intention recognition algorithm in Section 2, a ZNN-based controller is designed to realize the control of the upper limb rehabilitation robot without noise interference. Under the noise interference, to eliminate the noise interference of the upper limb rehabilitation robot in the actual rehabilitation training environment, a noise-suppressing zeroing neural network (NSZNN) model is designed to control the upper limb rehabilitation robot. The human upper limb skeleton diagram is displayed in Figure 5.

**Figure 6:**
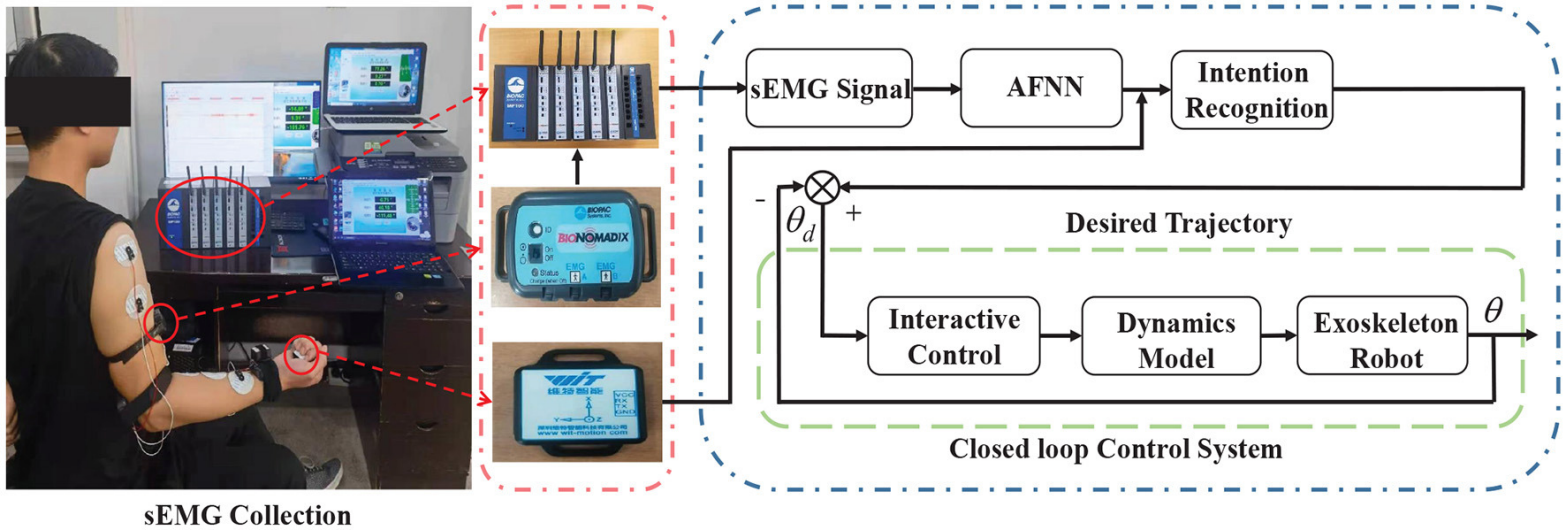
Technical flowchart of closed-loop control system.

The specific mathematical expression of the Lagrangian dynamics model of the upper limbs is:


(13) $$\begin{array}{l} M\left(\theta \right)\ddot{\theta }+C\left(\theta ,\overset{\cdot }{\theta }\right)\overset{\cdot }{\theta }+G\left(\theta \right)=\tau_{a} \end{array}$$

where *M*(θ) is the inertial matrix, $C\left(\theta ,\overset{\cdot }{\theta }\right)$is the centrifugal force and Coriolis force matrix, *G*(θ) is the gravity term matrix, $\overset{\cdot }{\theta }$ is the angle of the three joints of the upper limb, $\ddot{\theta }$ is the angular acceleration of the three joints of the upper limb, τ_*a*_ is the joint torque of the three joints during exercise. Based on the Lagrangian dynamics model (Equation 13) of human-machine coupling and the active movement intention of the upper limb, a human-machine interactive controller is designed to effectively control the upper limb rehabilitation. Under the premise of human-machine information interaction, rehabilitation robots can drive patients to perform rehabilitation training. The details of technical flowchart can be seen in Figure 6.

The control goal of the upper limb rehabilitation robot is to ensure that the error tends to zero. That is, the actual trajectory of the upper limb rehabilitation robot is close to the desired trajectory, and the error between the actual trajectory and the desired trajectory is close to zero. Based on the above analysis, the upper limb control problem is transformed into an time-varying zeroing finding problem, and the mathematical expression is as follows:


(14) $$\begin{array}{l} f\left(\chi \left(t\right),t\right)=0 \end{array}$$

where *f*(χ(*t*), *t*)∈*R*^*n*^ is a nonlinear mapping function. For ∀*t*∈[0, +∞), there is a corresponding χ(*t*)∈*R*^*n*^. Assuming that the theoretical solution is χ^*^(*t*) at this time, there is *f*(χ^*^(*t*), *t*) = 0 . At the same time, in order to ensure that the error function *e*(*t*) of the model converges to zero and χ(*t*) approaches its theoretical solution χ^*^(*t*), the key idea of the ZNN model is to establish the following error function:


(15) $$\begin{array}{l} e\left(t\right)=f\left(\chi^{*}\left(t\right),t\right)-f\left(\chi \left(t\right),t\right)=0 \end{array}$$

In order to ensure the error function (Equation 15) converges to zero, that is, at this time χ(*t*) converges to its theoretical solution x^*^(*t*), the zeroing dynamic model is established as:


(16) $$\begin{array}{l} ė\left(t\right)=-\gamma e\left(t\right) \end{array}$$

where γ as a positive real number, which can manually control its convergence rate.

Suppose χ(*t*) is a state variable, *u*(*t*) is a control input, and *e*(*t*) is an output of the nonlinear system. The (14) can be defined as the following classical nonlinear system:


(17) $$\{{}_{y(t)=f(\chi (t),t)=-e(t)}^{\dot{\chi }(t)=u(t)}$$

At this time, the time-varying controller can be obtained by analyzing and calculating (16) and (17) to ensure that the error function at this time converges to zero. Combining the error function with the zeroing neural dynamic model can get the following equation:


(18) $$\begin{array}{l} Z\left(\chi \left(t\right),t\right)\overset{\cdot }{\chi }\left(t\right)=-\left[\gamma f\left(\chi \left(t\right),t\right)+\frac{\partial f\left(\chi \left(t\right),t\right)}{\partial t}\right] \end{array}$$

where *Z*(χ(*t*), *t*) is a full rank matrix. Since the ZNN model cannot effectively suppress the noise interference in the rehabilitation process, and the integration technique is usually used in the control theory to deal with the noise interference, this paper introduces the integral term for (16) to obtain the following noise-suppression zeroing neural network (NSZNN) model:


(19) $$\begin{array}{l} ė(t)=-\gamma e(t)-\lambda \int_{0}^{t}e(s)\text{d}s \end{array}$$

where γ > 0, λ > 0, the convergence speed and noise suppression ability can be changed by adjusting the value of the two parameters. In fact, from a control viewpoint, the ZNN controller can be regarded as a general proportional-derivative (GPD) controller, and the NSZNN controller can be seemed as a general Proportional-integral-derivative (GPID) controller, which leverages time-derivative information for effective prediction and error-integration information for noise elimination. When the error function *e*(*t*) satisfies the condition of Equation (19), the state variable χ(*t*) of Equation (17) globally/exponentially converges to its theoretical solution x^*^(*t*), the NSZNN controller in the formula (Equation 17) can be expressed as follows:


(20) $$u(t)=-Z^{-1}(\chi (t),t)[\gamma f(\chi (t),t)+\lambda \int_{0}^{t}f(\chi (s),s)\text{d}s+\frac{\partial f(\chi (t),t)}{\partial t}]$$

The dynamic model of the upper limb is established as follows:


(21) $$\begin{array}{l} M\left(q_{R_{i}}\right){\ddot{q}}_{R_{i}}+C\left(q_{R_{i}},{\overset{\cdot }{q}}_{R_{i}}\right){\overset{\cdot }{q}}_{R_{i}}+G\left(q_{R_{i}}\right)=\tau_{i} \end{array}$$

where *M*(*q*_*R*_*i*__) represents the mass matrix, $C\left(q_{R_{i}},{\overset{\cdot }{q}}_{R_{i}}\right)$ represents the centripetal force and Coriolis force matrix, and *G*(*q*_*R*_*i*__) represents the gravity matrix. Combine the upper limb dynamics model with the ZNN controller and the NSZNN controller to obtain the upper limb controller is:


(22) $$\begin{array}{l} \tau_{i}^{\text{ZNN}}={\overset{\cdot }{\theta }}_{i}^{d}+\gamma \left(\theta_{i}^{d}-\theta_{i}\right) \end{array}$$

and


(23) $$\begin{array}{l} \tau_{i}^{\text{NSZNN}}=\tau_{i}^{\text{ZNN}}+\lambda \Sigma_{j=0}^{k}\left(\theta_{i}^{d}-\theta_{i}\right) \end{array}$$

where $\theta_{i}^{d}$ is the desired action angle, and θ_*i*_ is the actual action angle.

## 4. Experimental analyzes

In this section, some experimental examples simulate the human-machine interaction control of the upper limb rehabilitation robot. The implementation process of the human-machine interaction control for upper limb rehabilitation robot is displayed in Table 1. First, based on the sEMG signals of multiple muscles of the upper limbs, the multi-input multi-output AFNN model is used to identify the active movement intention of the human upper limbs shoulder joints, elbow joints and wrist joints. Second, the angle information of the different joints is calculated under the active movement intention based on the active movement intention and the human upper limb Lagrangian dynamic model. Besides, to verify the feasibility and efficiency of the proposed controller, some numerical results are described in this section. It is worth noting that the parameters of various models are set as follows. The parameters PID controller are set to *k*_*p*_ = 10, *K*_*i*_ = 0.5 and *K*_*d*_ = 0.2, respectively; The parameters of the NSZNN controller are set to γ = 100 and λ = 200, respectively. Similarly, The parameter of the ZNN controller and GNN controller are set to γ = 100.

**Table 1:** The implementation process of the human-machine interactive control for upper limb rehabilitation robot.

**Step 1**	**Multi-channel sEMG signal and multi-joint angle acquisition**
**Step 2**	**Processing of the raw sEMG signals (full-wave rectification, sub-sampling)**
**Step 3**	**Identify subjects' active motion intentions**
**Step 3.1**	**Input: the processed sEMG signals *x* = [*x*_1_, *x*_2_, ...*x*_*t*_]**
**Step 3.2**	**data normalization**
**Step 3.3**	**Building fuzzy neural network: $y=\Sigma_{j=1}^{L}f_{j}\phi_{j}\left(\frac{\left||x-\mu_{j}|\right|}{\sigma_{j}}\right)$**
**Step 3.4**	**Training network: while (i = 1, i < = maxstep & E>1e-10, i++)**
	**Modify the parameters (*f*_*j*_, *b*_*i, j*_, *c*_*i, j*_) in network**
	**end while**
**Step 3.5**	**Data inverse normalization**
**Step 3.6**	**Output: estimated multi-joint angle $y=\frac{\Sigma_{j}^{L}f_{j}\Pi_{i}^{n_{j}}e^{-\left(\frac{x_{i}-\mu_{ij}}{\sigma_{ij}}\right)}}{\Sigma_{j}^{L}\Pi_{i}^{n_{j}}e^{-\left(\frac{x_{i}-\mu_{ij}}{\sigma_{ij}}\right)}}$**
**Step 4**	**human-machine interaction control**
**Step 4.1**	**Desired trajectory: subjects' motion intention *y***
**Step 4.2**	**Building the Lagrangian dynamics model of the upper Limb:**
	** $M\left(\theta \right)\ddot{\theta }+C\left(\theta ,\overset{\cdot }{\theta }\right)\overset{\cdot }{\theta }+G\left(\theta \right)=\tau_{a}$ **
**Step 4.3**	**Design the human-machine interaction controller:**
	** $\tau_{i}^{\text{NSZNN}}={\overset{\cdot }{\theta }}_{i}^{d}+\gamma \left(\theta_{i}^{d}-\theta_{i}\right)+\lambda \Sigma_{j=0}^{k}\left(\theta_{i}^{d}-\theta_{i}\right)$ **
**Step 4.4**	**Determination of parameters γ>0, λ>0**
**Step 4.5**	**Solving nonlinear dynamic system with controller $\tau_{i}^{\text{NSZNN}}$**
	**if ||*e*||_2_ < μ**
	**Perform the next step (Step 4.6)**
	**else**
	**Return to the previous step (Step 4.4)**
	**end**
	**Return**
**Step 4.6**	**control sequence $\tau_{i}^{\text{NSZNN}}$ and the actual state trajectory θ_*i*_**

### 4.1. Experimental results without noise

**Figure 7:**
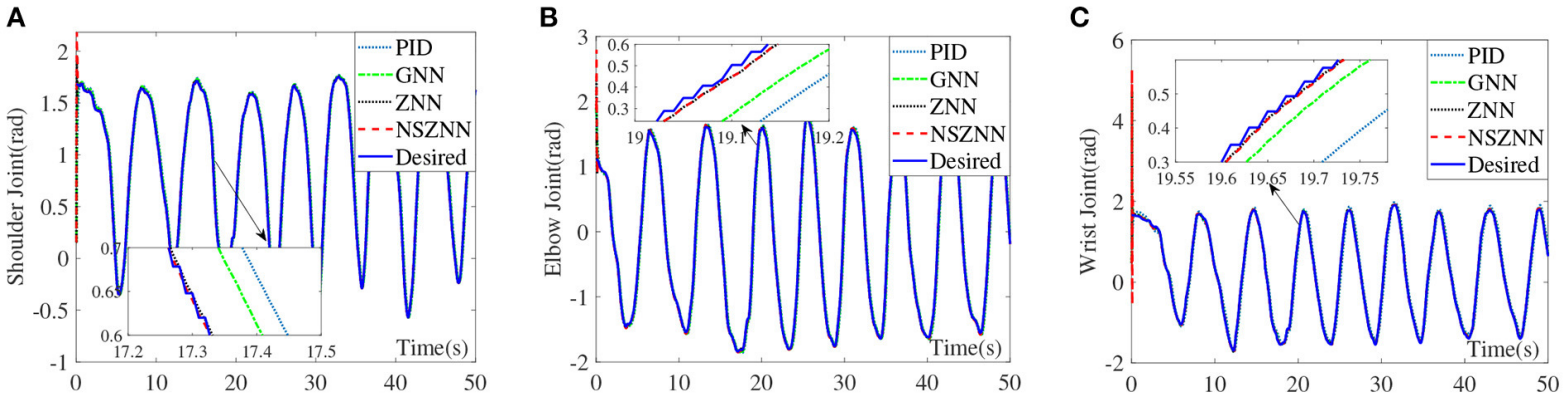
Comparison of the upper limb multi-joint angles without noise, **(A)** shoulder joint, **(B)** elbow joint, and **(C)** wrist joint.

In the absence of noise interference, the ZNN controller is used to effectively control the upper limb rehabilitation robot, and the NSZNN controller is used to effectively control the upper limb rehabilitation robot with different noises. In order to better show the performance of the model, the PID controller (), GNN controller (Zhang et al., 2009), ZNN controller and NSZNN controller are utilized to monitor the upper limb rehabilitation robot. By comparing the experimental errors of the four controllers, it shows that the NSZNN controller has the feasibility and superiority when used to control the upper limb rehabilitation robot. In addition, further research has been conducted on the adjustable parameters of the NSZNN controller, which achieves the characteristics of manual regulation. Under the same initial parameters, the designed NSZNN controller, ZNN controller, GNN controller and PID controller are exploited to monitor the upper limb shoulder, elbow, and wrist joint. The experimental results are shown in Figure 7.

It can be seen from the above experimental results that because of the initial value setting, the designed NSZNN controller, ZNN controller, GNN controller and PID controller all have large deviations within 0.5 s at the beginning, but as time reaches 1 s, the four kinds of controllers can track the trajectory of the three joints of the upper limbs. In addition, the average RMSEs of the PID controller, GNN controller, ZNN controller, and NSZNN controller are 0.0084, 0.0053, 0.0024, and 0.0008, respectively. It proves the total effect of the three joints under the control of NSZNN controller is not much different than that of the ZNN controller. Beyond that, the NSZNN controller and the ZNN controller have similar effects on the control of the three joints, and the RMSE value is much smaller than the GNN controller and PID controller. The accuracy of the NSZNN controller is increased by more than 85% and 90%compared with GNN controller and PID controller, respectively.

In the actual rehabilitation training process, the environment and other factors often cause corresponding interference to the rehabilitation process. In order to simulate the actual rehabilitation training process, constant noise, linear noise, and random noise are added to simulate the interference source.

### 4.2. Experimental results with constant noise

**Figure 8:**
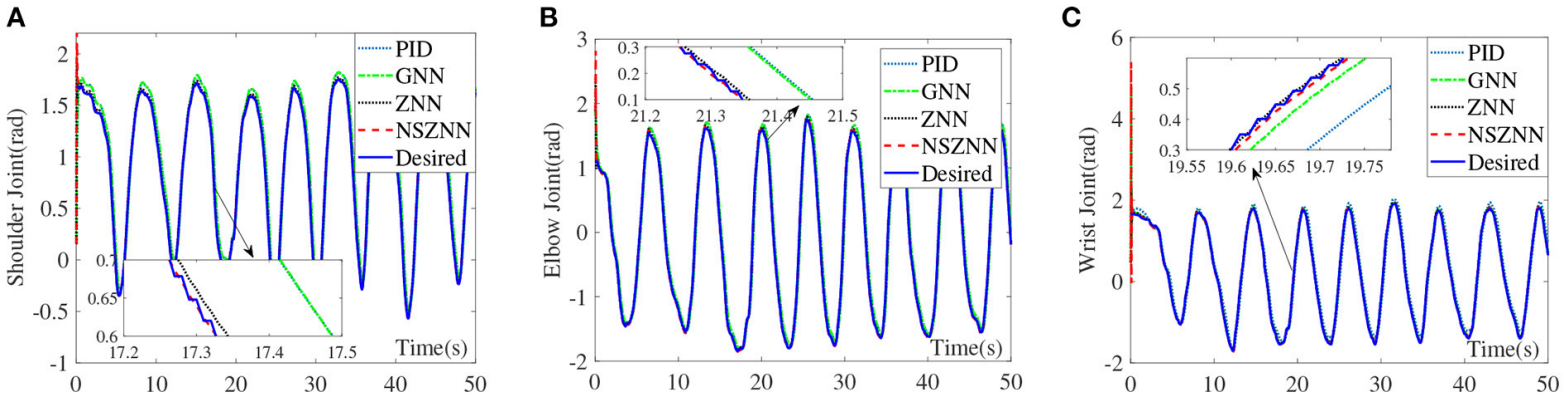
Comparison of the upper limb multi-joint angles with constant noise, **(A)** shoulder joint, **(B)** elbow joint, and **(C)** wrist joint.

With the constant noise, the control effect comparison diagram of the NSZNN controller, ZNN controller, GNN controller and PID controller on the three joints of the upper limb is shown in Figure 8.

Through the analysis of the above experimental results, it can be seen that the NSZNN controller, ZNN controller, GNN controller and PID controller with constant noise can achieve stable control of the three joints of the upper limb, which is consistent with the actual range of motion of the limb. But in the comparison of the angle of the shoulder joint, it can be clearly found that there are significant fluctuations in the GNN controller and PID controller for shoulder joint control at 0–5 s. However, as time goes by, the fluctuations gradually decrease and are consistent with the actual measured movement angle. Although the movement trend is in line with the actual movement trend of the shoulder joint, the GNN controller and PID controller has an obvious peak at the peak point; the designed NSZNN controller and ZNN controller can achieve accurate control of the shoulder joints, and there is no initial value fluctuation under GNN control, and the entire control process is relatively stable. And compared with ZNN controller, the NSZNN controller has the best tracking effect. Four controllers are relatively stable for the control of the elbow joint and the wrist joint, but there is a certain degree of excessive peak value under the GNN controller and PID controller. It can be clearly seen in the comparison chart of the control effect of the wrist joint that the NSZNN controller and ZNN controller, which can accurately track the desired trajectories. In addition, the NSZNN controller may perform better than the others. In fact, the GNN controller and PID controller has an obvious deviation. That is, the GNN controller and PID controller may not solve the problem with constant noise.

### 4.3. Experimental results with linear noise

**Figure 9:**
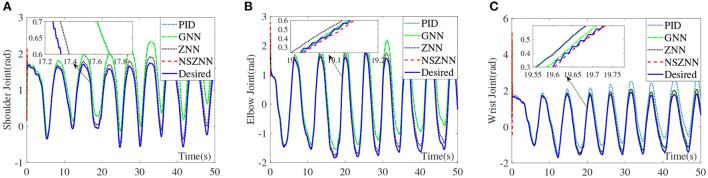
Comparison of the upper limb multi-joint angles with linear noise, **(A)** shoulder joint, **(B)** elbow joint, and **(C)** wrist joint.

Under the linear noise circumstance, the numerical results are shown in Figure 9. It can be seen that ZNN controller, GNN controller and PID controller have weaker ability to suppress linear noise, and both have obvious deviations. The NSZNN controller performs relatively stable during the entire trajectory tracking process. Through the comparison of the lower shoulder joint of the four controllers, it can be directly seen that the movement trend of the lower shoulder joint under GNN controller and PID controller is consistent with the actual range of shoulder joint motion. However, there is a large deviation in the tracking process, compared with the peak value of the shoulder joint with ZNN controller. The overall trend is consistent with the actual range of movement of the shoulder joint, and the NSZNN controller can track the desired trajectory more stably throughout the process. From the enlarged diagram of different figures, there is no large deviation compared to the ZNN controller and the GNN controller, and the trajectory is accurately achieved track. It can be found that the GNN controller and PID controller also exhibits a large deviation and the deviation becomes larger and larger over time, while the overall tracking effect of ZNN controller is relatively stable. Although the peak value is too high, the NSZNN controller performs best. Under the premise of accurate tracking, the whole process is relatively smooth, and there is no large degree of deviation. In other words, the NSZNN controller can effectively suppress linear noise, which infers that the NSZNN controller may be exploited to actual rehabilitation.

### 4.4. Experimental results and with random noise

**Figure 10:**
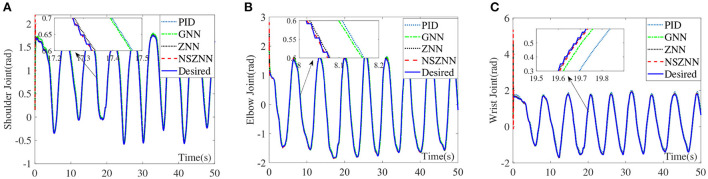
Comparison of the upper limb multi-joint angles with random noise, **(A)** shoulder joint, **(B)** elbow joint, and **(C)** wrist joint.

Under the random noise situation, the numerical results are shown in Figure 10. Although the four controllers can achieve stable tracking of the three-joint trajectory under random noise, it shows that the four controllers have a certain degree of suppression of random noise, it can be seen from the control effect of the shoulder joint that there is a small degree of excessive peak value under the GNN controller and PID controller. The ZNN controller and the NSZNN controller are relatively stable, which can be clearly found through the enlarged diagram that NSZNN controller and ZNN controller have higher accuracy and stability than GNN controller and PID controller. The NSZNN controller performs better than the ZNN controller under the random noise case. On the whole, the four controllers have achieved relatively stable tracking error for the elbow and wrist joints, and there is no significant fluctuation in the whole process. However, it can be clearly seen from the detailed enlarged view that the NSZNN controller and the ZNN controller can achieve trajectory tracking more accurately than GNN controller and PID controller, especially in the first 5 s, the NSZNN controller and the ZNN controller are more stable and the GNN controller and PID controller has a certain fluctuation.

### 4.5. Discussions and comparisons of different controllers

By comparing the angle tracking effects of the NSZNN controller, the ZNN controller, the GNN controller and the PID controller on the upper limb without noise, it can be seen that the NSZNN controller, ZNN controller, GNN controller and PID controller can complete relatively stable trajectory tracking. In order to simulate the interference in the actual limb rehabilitation training process, constant noise, linear noise, and random noise are added to simulate interference sources. The data in Table 2 further show that the four kinds of controllers have a certain degree of suppression of the three kinds of noises. Both the NSZNN controller and the ZNN controller have better noise-tolerant ability than the GNN controller and the PID controller, and the NSZNN controller has the best control effect. Among the three types of noise, the linear noise fluctuates greatly for the ZNN controller, the GNN controller and PID controller, while the overall performance of the NSZNN controllers are relatively stable. At this time, the RMSE of the ZNN controller is reduced by 2–3 times and 3–4 times compared with the GNN controller and PID controller, respectively. Beyond that, The NSZNN controller is increased by 2–10 times on the basis of the ZNN controller, which shows the excellent noise-suppression ability of the NSZNN controller. In addition, the NSZNN controller and the ZNN controller can achieve accurate trajectory tracking with the constant/random noise. The GNN controller and PID controller have relatively large deviations, and the noise suppression effect is worse than the traditional ZNN controller and the NSZNN controller. In other words, in practical application, the ZNN controller and the NSZNN controller can be used directly, which might have good noise-suppression ability. It may convenient for engineering implementation and clinical application.

**Table 2:** Comparison of three-joint RMSEs under different noises.

	**PID Controller**			**GNN Controller**			**ZNN Controller**			**NSZNN Controller**		

	**Shoulder**	**Elbow**	**Wrist**	**Shoulder**	**Elbow**	**Wrist**	**Shoulder**	**Elbow**	**Wrist**	**Shoulder**	**Elbow**	**Wrist**
Without noise	0.0113	0.0055	0.0085	0.0080	0.0025	0.0055	0.0034	0.0011	0.0026	0.0007	0.0009	0.0009
Constant noise	0.0739	0.0521	0.0652	0.0645	0.0489	0.0605	0.0184	0.0143	0.0179	0.0072	0.0045	0.0015
Linear noise	0.4531	0.4558	0.4552	0.4483	0.4357	0.4478	0.1278	0.1238	0.1276	0.0721	0.0040	0.0012
Random noise	0.0421	0.0312	0.0352	0.0385	0.0228	0.0342	0.0109	0.0067	0.0106	0.0053	0.0046	0.0016

**Figure 11:**
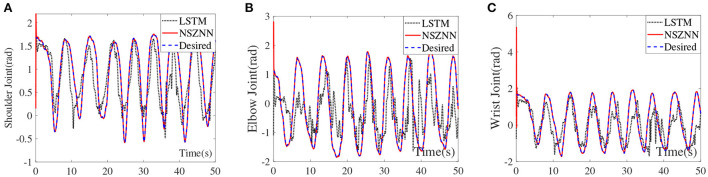
Compare NSZNN with LSTM of the multi-joint angles with random noise, **(A)** shoulder joint, **(B)** elbow joint, and **(C)** wrist joint.

Long short term memory (LSTM) network is an important neural network, which can be called as a recurrent neural network (RNN) (Bao et al., 2021). To demonstrate the effectiveness and superiority of the proposed NSZNN controller, numerical results are shown in Figure 11. The LSTM model consists of three LSTM layers, a dropout layer with 0.1 probability, five fully connected layers, and a softmax layer. The maximum number of iterations is set to 1,000. Figure 11 represent the shoulder joint angle, elbow joint angle and wrist joint angle of upper limb under the random noises *via* the NSZNN controller and the LSTM network controller, respectively. It can be inferred that the investigated NSZNN controller can effectively suppress the interference of random noises. Beyond that, the convergence and robustness of the NSZNN controller are superior to the LSTM model with random noise interference. In addition, to further compare the convergence ability of NSZNN controller and LSTM with different noises, the RMSE of NSZNN controller and LSTM model with different noises are calculated in this subsection, and the results are listed in Table 3. It can be seen from Table 3 that the NSZNN controller has better convergence performance than the LSTM model with different noises. Furthermore, the RMSEs of the NSZNN controller and the LSTM model with linear noise are correspondingly increased, which proves that the linear noise has the strongest interference on the model. However, the proposed NSZNN controller can still effectively suppress the interference of linear noise. The RMSEs of three joints are 0.0721, 0.0040, and 0.0012, respectively. It further illustrates the convergence and robustness of the developed NSZNN controller are superior to other traditional models.

**Table 3:** The RMSEs of the LSTM and the NSZNN models with different noises.

	**Constant noise**			**Random noise**			**Linear noise**		

	**Shoulder**	**Elbow**	**Wrist**	**Shoulder**	**Elbow**	**Wrist**	**Shoulder**	**Elbow**	**Wrist**
LSTM	0.1222	0.1031	0.1525	0.1497	0.1953	0.2671	0.4269	0.5880	0.6825
NSZNN	0.0072	0.0045	0.0015	0.0053	0.0046	0.0016	0.0721	0.0040	0.0012

## 5. Conclusions and future work

Aiming at the design of the human-machine interaction controller of the upper limb rehabilitation robot, this paper establishes a multi-input and multi-output AFNN model based on the sEMG signals to effectively predict the joint angles of the upper limb shoulder, elbow and wrist joints. Furthermore, to avoid secondary injury during the rehabilitation training process, a human-machine interaction controller based on the intention of human active movement is designed, which includes the ZNN controller and the NSZNN controller. It builds a safe, active and compliant rehabilitation training environment for patients. Experimental results prove that the ZNN controller is effective in tracking the real-time expected trajectory of the human upper limb shoulder joint, elbow joint and wrist joint without noise circumstance. In addition, considering that there are interfering factors such as joint damping, muscle contraction, and external friction in actual rehabilitation training, which brings the risk of secondary injury to the patient's rehabilitation training. The NSZNN controller is designed, which provides patients with a safe rehabilitation training environment. Experimental results demonstrate that the NSZNN controller is still feasible, effective and superior under different noise interference. Besides, the developed ZNN-based models will be exploited to the performance of the related applications, for instance, lower limb rehabilitation robot (Shi et al., 2020), industrial robot (Li et al., 2017), and redundant manipulators (Xie et al., 2022) and so on.

## Data availability statement

The raw data supporting the conclusions of this article will be made available by the authors, without undue reservation.

## Ethics statement

The studies involving human participants were reviewed and approved by Ethics Committee approval was obtained from the Institutional Ethics Committee of Medicine of the Second Hospital of Jilin University to the commencement of the study. The patients/participants provided their written informed consent to participate in this study. Written informed consent was obtained from the individual(s) for the publication of any potentially identifiable images or data included in this article.

## Author contributions

BZ and XL: data curation. BZ, XL, and XZ: conceptualization and formal analysis. GW, ZP, XZ, and ZS: methodology. GW and XZ: software. BZ, XL, and ZS: writing-original draft. GW, ZP, and ZS: supervision. GW, XZ, and ZS: validation. All authors have read and agreed to the published version of the manuscript.

## Funding

The work is supported in part by the National Natural Science Foundation of China, Grant Nos. 61873304 and 51875047; China Postdoctoral Science Foundation Funded Project, Grant Nos. 2018M641784 and 2019T120240; Key Science and Technology Projects of Jilin Province, China, Grant No. 20200201291JC, and Changchun Science and Technology Project, Grant No. 21ZY41.

## Conflict of interest

The authors declare that the research was conducted in the absence of any commercial or financial relationships that could be construed as a potential conflict of interest.

## Publisher's note

All claims expressed in this article are solely those of the authors and do not necessarily represent those of their affiliated organizations, or those of the publisher, the editors and the reviewers. Any product that may be evaluated in this article, or claim that may be made by its manufacturer, is not guaranteed or endorsed by the publisher.

## References

[B1] Aach C. M. Jansen O. Moisi M. Mayadev A. Pagarigan K. Dettori J.. (2016). The effectiveness and safety of exoskeletons as assistive and rehabilitation devices in the treatment of neurologic gait disorders in patients with spinal cord injury: a systematic review. Glob. Spine J. 6, 822–841. 10.1055/s-0036-1593805 27853668 PMC5110426

[B2] Bao T. Zaidi S. A. R. Xie S. Yang P. Zhang Z. Q. (2021). A cnn-lstm hybrid model for wrist kinematics estimation using surface electromyography. IEEE Trans. Instrum. Meas. 70, 1–9. 10.1109/TIM.2020.303665433776080

[B3] Brahim B. Saad M. Luna C. O. Rahman M. H. Brahmi A. (2018). Adaptive tracking control of an exoskeleton robot with uncertain dynamics based on estimated time-delay control. IEEE/ASME Trans. Mechatron. 23, 575–585. 10.1109/TMECH.2018.2808235

[B4] Cene V. H. Balbinot A. (2020). Resilient emg classification to enable reliable upper-limb movement intent detection. IEEE Trans. Neural Syst. Rehabil. Eng. 28, 2507–2514. 10.1109/TNSRE.2020.3024947 32956063

[B5] Chai Y. Y. Liu K. P. Li C. X. Sun Z. B. Jin L. Shi T. (2021). A novel method based on long short term memory network and discrete-time zeroing neural algorithm for upper-limb continuous estimation using semg signals. Biomed. Signal Process. Control 67, 102416. 10.1016/j.bspc.2021.102416

[B6] Chen S. H. Lien W. M. Wang W. W. Lee G. D. Hsu L. C. Lee K. W.. (2016). Assistive control system for upper limb rehabilitation robot. IEEE Trans. Neural Syst. Rehabil. Eng. 24, 1199–1209. 10.1109/TNSRE.2016.2532478 26929055

[B7] Deng M. Z. Li Y. Kang C. L. Chen P. (2020). A learning-based hierarchical control scheme for an exoskeleton robot in human-robot cooperative manipulation. IEEE Trans. Cybern. 50, 112–125. 10.1109/TCYB.2018.2864784 30183653

[B8] Fournier B. N. Lemaire E. D. Smith A. J. J. Doumit M. (2018). Modeling and simulation of a lower extremity powered exoskeleton. IEEE Trans. Neural Syst. Rehabil. Eng. 26, 1596–1603. 10.1109/TNSRE.2018.2854605 30004879

[B9] Han J. D. Ding Q. C. Xiong A. B. Zhao X. G. (2015). A state-space emg model for the estimation of continuous joint movements. IEEE Trans. Ind. Electron. 62, 4267–4275. 10.1109/TIE.2014.238733728113324

[B10] Huang Y. J. Chen K. B. Zhang X. M. Wang K. Ota J. (2021). Motion estimation of elbow joint from semg using continuous wavelet transform and back propagation neural networks. Biomed. Signal Process. Control. 68, 102657. 10.1016/j.bspc.2021.102657

[B11] Huo W. Mohammed S. Moreno J. C. Amirat Y. (2016). Lower limb wearable robots for assistance and rehabilitation: a state of the art. IEEE Syst. J. 10, 1068–1081. 10.1109/JSYST.2014.235149135768486

[B12] Jin L. Li S. Hu B. (2018). Rnn models for dynamic matrix inversion: a control-theoretical perspective. IEEE Trans. Ind. Inform. 14, 189–199. 10.1109/TII.2017.2717079

[B13] Jin L. Li S. Hu B. Liu M. Yu J. (2019). Noise-suppressing neural algorithm for solving time-varying system of linear equations: a controlbased approach. IEEE Trans. Ind. Inform. 15, 236–246. 10.1109/TII.2018.2798642

[B14] Jin L. Zhang Y. N. Li S. Zhang Y. Y. (2016). Modified znn for time-varying quadratic programming with inherent tolerance to noises and its application to kinematic redundancy resolution of robot manipulators. IEEE Trans. Ind. Electron. 63, 6978–6988. 10.1109/TIE.2016.2590379

[B15] Jin L. Zhang Y. N. Li S. Zhang Y. Y. (2017). Noise-tolerant znn models for solving time-varying zero-finding problems: a control-theoretic approach. IEEE Trans. Automat. Contr. 62, 992–997. 10.1109/TAC.2016.2566880

[B16] Kim S. Bae J. (2016). “Force-mode control of rotary series elastic actuators in a lower extremity exoskeleton using model-inverse time delay control (MiTDC),” in 2016 IEEE/RSJ International Conference on Intelligent Robots and Systems (IROS) (Daejeon: IEEE), 3836–3841.

[B17] Li C. Yang C. Wan J. Annamalai A. Cangelosi A. (2017). “Neural learning and kalman filtering enhanced teaching by demonstration for a baxter robot,” in 2017 23rd International Conference on Automation and Computing (ICAC) (Huddersfield), 1–6.

[B18] Liu H. Tao J. Lyu P. Tian F. (2020). Human-robot cooperative control based on semg for the upper limb exoskeleton robot. Rob. Auton. Syst. 125, 103350. 10.1016/j.robot.2019.103350

[B19] Nougarou F. Campeau-Lecours A. Massicotte D. Boukadoum M. Gosselin C. Gosselin B. (2019). Pattern recognition based on hd-semg spatial features extraction for an efficient proportional control of a robotic arm. Biomed. Signal Process. Control 53, 101550. 10.1016/j.bspc.2019.04.027

[B20] Orekhov G. Fang Y. Luque J. Lerner Z. F. (2020). Ankle exoskeleton assistance can improve over-ground walking economy in individuals with cerebral palsy. IEEE Trans. Neural Syst. Rehabil. Eng. 28, 461–467. 10.1109/TNSRE.2020.2965029 31940542 PMC7050636

[B21] Park Y. Paine N. Oh S. (2018). Development of force observer in series elastic actuator for dynamic control. IEEE Trans. Ind. Electron. 65, 2398–2407. 10.1109/TIE.2017.274545735161799

[B22] Peng G. Z. Yang C. G. He W. Chen C. L. P. (2020). Force sensorless admittance control with neural learning for robots with actuator saturation. IEEE Trans. Ind. Electron. 67, 3138–3148. 10.1109/TIE.2019.2912781

[B23] Shi T. Tian Y. T. Sun Z. B. Zhang B. C. Pang Z. X. Yu J. Z.. (2020). A new projected active set conjugate gradient approach for taylor-type model predictive control: application to lower limb rehabilitation robots with passive and active rehabilitation. Front. Neurorobot. 14, 559048. 10.3389/fnbot.2020.559048 33343324 PMC7744727

[B24] Sun Z. B. Shi T. Wei L. Sun Y. Y. Liu K. P. Jin L. (2020). Noise-suppressing zeroing neural network for online solving time-varying nonlinear optimization problem: a control-based approach. Neural Comput. Appl. 32, 11505–11520. 10.1007/s00521-019-04639-2

[B25] Sun Z. B. Wang G. Jin L. Cheng C. Zhang B. C. Yu J. Z. (2022). Noise-suppressing zeroing neural network for online solving time-varying matrix square roots problems: a control-theoretic approach. Expert. Syst. Appl. 192, 116272. 10.1016/j.eswa.2021.116272

[B26] Teng L. Gull M. A. Bai S. (2020). Pd-based fuzzy sliding mode control of a wheelchair exoskeleton robot. IEEE/ASME Trans. Mechatron. 25, 2546–2555. 10.1109/TMECH.2020.2983520

[B27] Venkatesh K. Shounak K. G. Madhubanti M. Geetha M. Dwaipayan S. (2019). Spinal cord injury: pathophysiology, treatment strategies, associated challenges, and future implications. Cell Tissue Res. 377, 125–151. 10.1007/s00441-019-03039-1 31065801

[B28] Wang G. Liu Y. B. Shi T. Duan X. Q. Liu K. P. Sun Z. B.. (2019). “A novel estimation approach of semg-based jointmovements via rbf neural network,” in 2019 Chinese Automation Congress (CAC) (Hangzhou), 1783–1788.

[B29] Wei P. Zhang J. H. Tian F. F. Hong J. (2021). A comparison of neural networks algorithms for eeg and semg features based gait phases recognition. Biomed. Signal Process. Control 68, 102587. 10.1016/j.bspc.2021.102587

[B30] Wu Q. C. Wang X. S. Bai C. Wu H. (2018a). Development of an rbfn-based neural-fuzzy adaptive control strategy for an upper limb rehabilitation exoskeleton. Mechatronics 53, 85–94. 10.1016/j.mechatronics.2018.05.014

[B31] Wu W. Fong J. Crocher V. Peter V. S. L. Oetomo D. Tan Y.. (2018b). Modulation of shoulder muscle and joint function using a powered upper-limb exoskeleton. J. Biomech. 72, 7–16. 10.1016/j.jbiomech.2018.02.019 29506759

[B32] Xie Z. T. Jin L. Luo X. Sun Z. B. Liu M. (2022). Rnn for repetitive motion generation of redundant robot manipulators: an orthogonal projection-based scheme. IEEE Trans. Neural Netw. Learn. Syst. 33, 615–628. 10.1109/TNNLS.2020.3028304 33079680

[B33] Young A. J. Ferris D. P. (2017). State of the art and future directions for lower limb robotic exoskeletons. IEEE Trans. Neural Syst. Rehabil. 25, 171–182. 10.1109/TNSRE.2016.2521160 26829794

[B34] Zhang F. Li P. Hou Z.-G. Lu Z. Chen Y. Li Q.. (2012). semg-based continuous estimation of joint angles of human legs by using bp neural network. Neurocomputing 78, 139–148. 10.1016/j.neucom.2011.05.033

[B35] Zhang J. J. Cheah C. C. (2015). Passivity and stability of human-robot interaction control for upper-limb rehabilitation robots. IEEE Trans. Robot. 31, 233–245. 10.1109/TRO.2015.2392451

[B36] Zhang Y. N. Chen K. Tan H. Z. (2009). Performance analysis of gradient neural network exploited for online time-varying matrix inversion. IEEE Trans. Automat. Contr. 54, 1940–1945. 10.1109/TAC.2009.2023779

[B37] Zhao Y. Paine N. Jorgensen S. J. Sentis L. (2018). Impedance control and performance measure of series elastic actuators. IEEE Trans. Ind. Electron. 65, 2817–2827. 10.1109/TIE.2017.274540

